# Implications of the clinical gestational diagnosis of ZIKV infection in the manifestation of symptoms of postpartum depression: a case-control study

**DOI:** 10.1186/s12888-019-2157-9

**Published:** 2019-06-26

**Authors:** Eleomar Vilela Moraes, Olegário Rosa Toledo, Flávia Lúcia David, Bruna Nascimento Godoi, Keila Araujo Monteiro, Thaisa Cimardi Deluqui, Thais Wérica Teixeira, Andiara Luiza Carvalho, Mariza Martins Avelino

**Affiliations:** 1 School of Medicine, Federal University of Goiás– UFG, Av. Primeira Avenida, s/n, Setor Leste Universitário, Goiânia, GO 74605-020 Brazil; 2 Institute of Biological Sciences and Health, Federal University of Mato Grosso, UFMT, BR 070, Km 05, Barra do Garças, MT, 78600-000 Brazil; 3 Institute of Biological Sciences and Health, Pharmacy Course, Federal University of Mato Grosso, UFMT, BR 070, Km 05, Barra do Garças, MT, 78600-000 Brazil; 4 Department of Pediatrics and Child Care, School of Medicine, Federal University of Goiás, UFG, Av. Primeira Avenida, s/n, Setor Leste Universitário, Goiânia, GO 74605-020 Brazil; 5 Avenida Getúlio Vargas, 663, Aragarças, GO, 76240-000 Brazil

**Keywords:** Pregnancy, Puerperium, Women, Divorce, EPDS

## Abstract

**Background:**

Traumatic events can trigger postpartum depression. Pregnant women in Brazil today are facing an extremely stressful experience. Thus, the objective here was to analyze the prevalence of symptoms of depression in the immediate postpartum period (IPPD) and associate these symptoms with previous stressful, social, psychological, behavioral, obstetrical, clinical, violent and infectious events, particularly exposure to Zika virus (ZIKV) infection during pregnancy.

**Methods:**

This was a case-control study. The sample comprised 213 puerperal women treated in the maternity ward of a public reference hospital in the Araguaia River Valley of Mato Grosso and Goiás, Brazil. Severe IPPD symptoms were confirmed based on the Edinburgh Postnatal Depression Scale (EPDS). A descriptive statistical analysis was carried out using the Epi Info™ version 7.1.5 suite of software tools and the Statistical Package for Social Sciences (SPSS) version 21.0.

**Results:**

A bivariate analysis revealed a significant association between “severe symptoms of IPPD” and the following variables: “clinical diagnosis of ZIKV during pregnancy” (OR = 13.36; 95% CI = 5.34–33.39); “was separated or divorced in the last year” (OR = 3.58; 95% CI = 1.42–8.99); “suffered an accident in the last year” (OR = 3.32, 95% CI = 1.12–9.82); “suffered emotional violence during pregnancy” (OR = 3.80; 95% CI = 1.81–7.99); “suffered physical violence during pregnancy” (OR = 11.86; 95% CI = 2.07–67.82); “fear of her partner” (OR = 17.90; 95% CI = 3.44–92.99); “dengue fever during pregnancy” (OR = 7.85; 95% CI: 1.66–37.05), and “has a family member diagnosed with mental illness” (OR = 2.54; 95% CI = 1.09–5.93). The multivariate analysis confirmed the association of severe PPD symptoms only with the variables of “clinical diagnosis of ZIKV during pregnancy” (OR = 19.82; 95% CI: 5.35–73.39) and “was separated or divorced in the last year” (OR = 3.92; 95% IC = 1.12–13.63).

**Conclusions:**

Clinically diagnosed ZIKV during pregnancy may be one of the main events associated with severe IPPD symptoms, showing an almost 20-fold higher chance of occurrence than other factors.

## Introduction

The World Health Organization (WHO, 2012) [1] states that unipolar depressive disorders will be the leading cause of women’s illness worldwide by 2030. Postpartum depression (PPD) is a non-communicable depressive disorder that can occur up to 1 year postpartum [2]. The prevalence of PPD is approximately 19.8% in developing countries [3] and 26.3% in Brazil [4]. Albeit treatable, PPD causes widespread suffering among mothers and their babies, with negative effects on the entire family. The basic symptoms of PPD are sadness, anxiety, low self-esteem, decreased concentration and social contact, and they may coexist with different somatic disorders and even panic attacks [5].

A recent study involving 23,894 Brazilian puerperal women confirmed several risk factors for postpartum depression, such as low income, history of mental disorder, unplanned pregnancy and multiparity [4]. The literature also describes other predictive factors, such as marital conflicts, anxiety [6, 7], genetic factors, exposure to violence [1], history of emotional problems [8], diseases during pregnancy [3] and traumatic and/or stressful events [3, 7, 9].

Pregnant women in Brazil today are facing an extremely stressful experience that is a grave concern for the world population. The country is experiencing an outbreak of Zíka virus (ZIKV) transmitted by the mosquito *Aedes* (*A. aegypti* and *A. albopictus*). Based on several studies, the CDC confirmed that pregnant women can transmit ZIKV to their fetus throughout gestation, with the risk of causing microcephaly and other brain defects [10]. According to Brazil’s Ministry of Health (MS), 13,719 suspected cases have been diagnosed since the beginning of the outbreak [11].

In this context, several studies have shown that traumatic events can trigger PPD [7, 9], predisposing those who suffer from this disorder to adopt a less healthy lifestyle, with a history of risky attitudes [12, 13] that lead to poor health [14]. Therefore it can be stated that pregnant women in Brazil, who are potentially vulnerable to ZIKV, have become emotionally affected.

Thus, the objective here was to analyze the exposure to clinically diagnosed ZIKV infection during pregnancy and its association with symptoms of IPPD.

## Methods

An unpaired case-control study was carried out from April 2016 to August 2017 to evaluate exposure to clinically diagnosed ZIKV infection during pregnancy in puerperal patients ready to be discharged from hospital. These women were patients in the maternity ward of a public reference hospital in Barra do Garças, state of Mato Grosso (MT), which serves the Araguaia River Valley of Mato Grosso and Goiás, Brazil.

The sample was probabilistic and involved puerperal women 18 years or older. Women displaying symptoms of depression (EPDS ≥13) in the immediate postpartum period (2nd or 3rd day postpartum), exposed or not to ZIKV, were considered “cases,” while puerperal women without symptoms of depression (EPDS < 13), exposed or not to ZIKV, were considered “controls.”

The sample size was calculated using OpenEpi version 3.01 software [15]. A frequency of exposure of 67% was predicted among the cases [16], and of 40% among the controls, with a detection power (1-β) of 80%, confidence level of 95%, and a case-to-control ratio of one to five. The minimum number obtained was 40 cases and 119 controls, with a sample size of 159 puerperal women [17]. Given the possibility of losses, this number was increased by 34%, making a total of 213 respondents.

Data were collected on alternate days, when half of the puerperal women were invited to participate. It was decided to divide the study into segments for easier understanding. An investigation was made into the probable relationship between PPD symptoms and several variables known to be correlated with this disorder (social, economic, cultural, demographic, psychological, behavioral, stressful and obstetric characteristics, history of violence, and clinical and infectious diseases).

The first segment consisted of screening depression symptoms based on the Edinburgh Postnatal Depression Scale (EPDS), whose questions are structured, closed and standardized for epidemiological studies. The EPDS was originally developed by Cox and Holden [18], and the Portuguese version of the scale, which was devised by Santos et al. [19], consists of 10 questions, each containing four score options, from 0 to 3, with a maximum score of 30. The cut-off point used for the diagnosis of symptoms of depression was ≥13, which is specific for a high-risk population and ensures 59.5% sensitivity and 88.4% specificity [19].

Cases of violence against pregnant women were also screened using the Abuse Assessment Screen (AAS). This tool was developed in the USA [20] and has been validated in Brazil [21]. The abridged version of the tool has five closed and open questions that identify the persistence and seriousness of the physical abuse, the parts of the body injured on a specific occasion, and the profile of the aggressor. The AAS has proved to be a suitable tool for the detection of violence against pregnant women [22, 23].

The second segment consisted of investigating several stressful events experienced by puerperal women, such as behavior, obstetric complications during the current pregnancy, and previous psychiatric history of the pregnant woman and her family.

The third segment involved the social, economic, cultural and demographic characteristics, assessing the location of residence, level of education, marital status, occupation and socioeconomic status. A low level of education was defined as less than or up to 8 years of schooling, and the socioeconomic status was determined based on the criteria proposed by the Brazilian Market Research Association (ABEP) [24]. The last two segments of the questionnaire were developed by the research team, which used bold font for explanatory notes to facilitate filling out, applying and typing. This part of the questionnaire was validated in a pilot study.

The pilot study was previously applied to 15 puerperal women in order to detect not only possible errors in the investigation but also ambiguities and difficulties in understanding the questions composed by the authors of this study. After identifying all possible errors, the necessary corrections were made.

The reliability of the EPDS scale was checked by calculating Cronbach’s alpha, according to which, the higher the coefficient the more reliable the instrument [25]. Thus, a coefficient above 0.70 was adopted as suitable for this study [26].

Excluded from this study were illiterate puerperal women getting treated for depression, women who failed to answer all the EPDS questions, and women with a previously diagnosed neurological disorder.

The data were evaluated using SPSS version 20 software (Statistical Package for Social Sciences). The variables were analyzed statistically using measures of central tendency (mean and median), dispersion (standard deviation) and frequency. Associations were verified based on a bivariate analysis using the χ2 test, Mantel-Haenszel test, Fisher’s Exact test and the Odds Ratio (OR), while logistic regression was used for the multivariate analysis. The input data were of the hierarchical type and the explanatory variables were based on the borderline *p*-value (*p* ≤ 0.10) and on clinical importance [27]. A linear regression analysis was performed to identify possible multicollinearity between the independent variables [28, 29]. In all the tests, a p-value lower than or equal to 5% (*p* ≤ 0.05) was considered to be of statistical significance within a 95% confidence interval. The main independent variable was a “clinically diagnosed ZIKV infection during pregnancy.” This diagnosis was confirmed based on the notification report issued by the Department of Health of the city of Barra do Garças, MT. “IPPD symptoms” was defined as a dependent variable.

This study was approved by the Research Ethics Committee (CEP) of the Federal University of Mato Grosso (UFMT) under Protocol CEP/UFMT/2015 No. 975.413, in line with Resolution No. 466/12 of the National Health Council of the Brazilian Ministry of Health.

## Results

Among the puerperal women with IPPD, 11.27% exhibited mild or moderate symptoms of depression (10 to 12 points) and 16.90% displayed severe symptoms (13+ points). As for the internal consistency of the total dataset, Cronbach’s alpha for the EPDS was 0.82, indicating the high reliability of the measurements (Table 1).

**Table 1:** Description of IPPD symptoms, Brazil, 2017 (*n* = 213)

EPDS^a^ (10 items)	n (%)	Cronbach’s Alpha	Interval	Median	Mean (SD)
		0.82	0–27	6	7.06 (5.54)
No IPPD (0–9 points)	153	71.83			
Mild/Moderate IPPD (10–12 points)	24	11.27			
Severe IPPD (13+ points)	36	16.90			

*SD* Standard deviation; ^a^Score of ≥13 on the *EPDS* Edinburgh postnatal depression scale

The interviewees ranged in age from 18 to 45 (25 ± 5.51). Most of them had more than 8 years of schooling (75.12%), a husband or partner (75.59%), a monthly income of more than three minimum salaries (47.00%), were self-employed (69.00%), and had a formally employed partner (50.70%). Furthermore, a significant number of them identified themselves racially as black (73.24) and lived in the outskirts, i.e., the poorest neighborhoods, of urban areas (86.85%). It was also found that 51.64% of them had not traveled during their pregnancy. The bivariate analysis revealed no statistically significant association between the variables.

Upon analyzing the history of stressful events, a significant association was found between a patient’s IPPD symptoms and her separation or divorce in the last year (OR = 3.58), a clinical diagnosis of ZIKV infection during pregnancy (OR = 13.36), and an accident in the last year (OR = 3.32). Surprisingly, however, this disorder was not influenced by any behavioral aspect (Table 2).

**Table 2:** Behavioral characteristics and stressful events pertaining to the health of puerperal women, Brazil, 2017 (*n* = 213)

Characteristics	PPD Symptoms^a^				Statistics	
	Cases		Controls		OR (CI 95%)	p*
	n	%	n	%		
Behavioral variable ^b^						
Consumption of alcohol beverages during pregnancy						
Yes	6	18.18	27	81.82	1.14(0.43–3.01)	0.989*
No	29	16.29	149	83.71		
Cigarette smoker						
Yes	9	20.00	36	80.00	1.38(0.59–3.21)	0.603*
No	25	15.34	138	84.66		
User of marijuana, cocaine, freebase or crack before pregnancy						
Yes	3	27.27	8	72.73	1.97(0.50–7.82)	0.397**
No	32	16.00	168	84.00		
Was in contact with chemicals (pesticides) during pregnancy						
Yes	7	25.00	21	75.00	1.78(0.69–4.57)	0.347*
No	29	15.76	155	84.24		
History of stressful events^b^						
Clinical diagnosis of ZIKV during pregnancy						
Yes	16	61.54	10	38.46	13.36(5.34–33.39)	0.001*
No	20	10.70	167	89.30		
Separated or divorced in the last year						
Yes	9	37.50	15	62.60	3.58(1.42–8.99)	0.011*
No	27	14.36	161	85.64		
Lost her job in the last year						
Yes	5	18.52	22	81.48	1.13(0.40–3.21)	0.787**
No	31	16.76	154	83.24		
Was involved in a lawsuit in the last year						
Yes	6	31.58	13	68.42	2.51(0.88–7.11)	0.103**
No	30	15.54	163	84.46		
Someone close to her died in the last year						
Yes	10	17.54	47	82.46	1.10(0.49–2.46)	0.985*
No	25	16.23	129	83.77		
Was assaulted in the last year						
Yes	0	0.00	1	100.00	1.20(1.130–1.275)	1.000**
No	35	16.67	175	83.33		
A thief broke into her home in the last year						
Yes	0	0.00	9	100	1.21(1.136–1.288)	0.361**
No	35	17.33	167	82.67		
Suffered an accident in the last year						
Yes	6	37.50	10	62.50	3.32(1.12–9.82)	0.035**
No	30	15.31	166	84.69		

*OR* Odds ratio, *CI* Confidence interval; χ2 Test; *Mantel-Haenszel test;** Fisher’s Exact test; ^a^Score of ≥13 on the *EPDS* Edinburgh postnatal depression scale; ^b^Some did not respond

An analysis of the history of violence (Table 3) revealed a significant association between PPD symptoms and the variables of “emotional violence during pregnancy” (OR = 3.80); “physical violence during pregnancy” (OR = 11.86) and “fear of her partner” (OR = 17.90).

**Table 3:** Evaluation of severe symptoms of IPPD attributed to violence suffered during pregnancy, Brazil, 2017 (*n* = 213)

Characteristics	PPD Symptoms^a^				Statistics	
	Cases		Controls		OR(CI 95%)	*p*
	n	%	n	%		
History of violence^b^						
Emotional violence during pregnancy						
Yes	19	32.20	40	67.80	3.80(1.81–7.99)	0.001*
No	17	11.11	136	88.89		
Physical violence during pregnancy						
Yes	4	66.67	2	33.33	11.86(2.07–67.82)	0.007**
No	28	14.48	166	85.57		
Sexual abuse in the last year						
Yes	1	50.00	1	50.00	5.21(0.32–85.43)	0.302**
No	33	16.10	172	83.90		
Fear of her partner						
Yes	6	75.00	2	25.00	17.90(3.44–92.99)	0.001**
No	29	14.36	173	85.64		
Fear of someone else						
Yes	3	33.33	6	66.67	2.68(0.64–11.28)	0.170**
No	31	15.74	166	84.26		

*OR* Odds ratio, *CI* Confidence interval; χ2 test; *Mantel-Haenszel test;** Fisher’s Exact test; ^a^Score of ≥13 on the *EPDS* Edinburgh postnatal depression scale; ^b^Some did not answer

As can be seen in Table 4, an association was found between PPD symptoms and puerperal women who had a family member with a diagnosis of mental disorder (OR = 2.54) and dengue fever during pregnancy (OR = 7.85).

**Table 4:** Evaluation of clinical and obstetric history of severe symptoms of IPPD, Brazil, 2017 (*n* = 213)

Characteristics	PPD Symptoms^b^				Statistics	
	Cases		Controls		OR (CI 95%)	p*
	n	%	n	%		
Clinical history^a^						
Dengue fever during pregnancy						
Yes	4	57.14	3	42.86	7.85(1.66–37.05)	0.014**
No	27	14.52	159	85.48		
Diagnosis of a STD						
Yes	3	30.00	7	70.00	2.19(0.54–8.93)	0.379**
No	33	16.34	169	83.66		
Family member diagnosed with a mental disorder						
Yes	12	25.53	35	74.47	2.54(1.09–5.93)	0.050*
No	15	11.90	111	88.10		
Obstetric variables ^a^						
Multiparous	27	20.45	105	79.55	2.31(1.00–5.38)	0.073*
Primiparous	8	10.00	72	90.00		
Current pregnancy was planned						
Yes	21	16.03	110	83.97	0.90(0.40–2.01)	0.964*
No	11	17.46	52	82.54		
Induced abortion						
Yes	2	66.67	1	33.33	10.42(0.92–118.31)	0.123**
No	33	16.10	172	83.90		
Spontaneous abortion						
Yes	8	17.178	37	82.22	1.09(0.46–2.60)	0.974*
No	27	16.56	136	83.44		
Risky pregnancy						
Yes	11	21.57	40	78.43	1.60(0.72–3.57)	0.347*
No	23	14.65	134	85.35		
Complications during pregnancy						
Yes	18	19.15	76	80.85	1.30(0.61–1.09)	0.630*
No	14	15.38	77	84.62		
Prenatal care						
No	9	26.47	25	73.53	1.99(0.84–4.72)	0.185*
Yes	27	15.34	149	84.66		
Cesarean section	24	15.48	131	84.52	0.81(0.36–1.82)	0.760*
Vaginal delivery	10	18.52	44	81.48		

*OR* Odds ratio, *CI* Confidence interval; χ2 test; *Mantel-Haenszel test;** Fisher’s Exact test ^a^Some did not answer; ^b^Score of ≥13 on the *EPDS* Edinburgh postnatal depression scale

Lastly, Table 5 describes a multivariate analysis of the variables, unlike the preceding tables, which describe the bivariate analysis. Among all the evaluated characteristics, only puerperal women with a “clinical diagnosis of ZIKV infection during pregnancy” (OR = 19.82) and women divorced or separated from their partner in the last year (OR = 3.92) showed a confirmed association with the symptoms of IPPD. Multicollinearity between the independent variables was found to be weak, since the Variance Inflation Factor (VIF) varied from 1.12 to 1.14 [29].

**Table 5:** Logistic regression analysis of events associated with severe symptoms of IPPD, Brazil, 2017 (*n* = 213)

Characteristics ^a^	Statistics	
	OR (CI 95%)	*P*
Fear of her partner	6.17(0.50–75.30)	0.154
Clinical diagnosis of ZIKV infection during pregnancy	19.82(5.35–73.39)	0.001
Physical violence during pregnancy	0.98(0.80–12.148)	0.990
Dengue fever during pregnancy	3.74(0.42–33.23)	0.237
Emotional violence during pregnancy	1.98(0.69–5.67)	0.201
Separated or divorced in the last year	3.92(1.12–13.63)	0.032
Had an accident in the last year	1.73(0.37–8.08)	0.485

*OR* Odds ratio, *CI* Confidence interval; ^a^The total characteristics may vary due to lack of information; Score of ≥13 in the *EPDS* Edinburgh postnatal depression scale

## Discussion

This may be the first study that investigates the association between IPPD and the clinical diagnosis of ZIKV infection in Brazil. It should be noted that, after adjusting the confounding variables, the episodes of “separation or divorce in the last year” (OR = 3.72) and “clinical diagnosis of ZIKV infection during pregnancy” were associated with the onset of IPPD symptoms (OR = 19, 82).

It should be kept in mind that women treated at private hospitals and women treated for psychiatric disorders, among which mood disorders were common, were excluded from the study. Therefore, it is quite possible that IPPD symptoms are underestimated or underreported. On the other hand, results similar to those of this survey were reported in a Brazilian article, which used the same scale and cutoff point [30].

With regard to the instrument, the EPDS scale was adopted chosen due to its applicability, since it was validated in an equivalent population [19] and has been used in numerous studies on pregnancy and puerperium. A cut-off point of ≥13 instead of ≥10 was chosen in order to exclude false positives and eliminate patients with milder symptoms, thereby avoiding a conflicting diagnosis of “melancholia in motherhood” or “baby blues,” which determines mild symptoms of depression immediately post delivery [19]. Moreover, this cutoff point is more favorable for diagnosing PPD symptoms in high-risk situations, preventing the loss of such women. It should be noted that the EPDS refers to the occurrence of PPD symptoms within a period of 7 days postpartum, and therefore does not exclude the possibility that this condition began during pregnancy [31–33].

In this study, no correlation was found between social, demographic and economic status and PPD symptoms, possibly because most of the women were married, were on average 25 years old, had more than 8 years of schooling, and their husbands were formally employed. Thus, it is reasonable to infer that this population enjoyed a stable social and economic status. These data are corroborated by the findings of Abdollahi et al. [34], who reported a positive association between advanced maternal age, low educational level [8], low family income and risk of PPD [34]. Moreover, another study found a correlation between the marital status of single or separated and illness, and that married women were more protected [33].

As for possible risks to mother and baby health, stressful episodes should be carefully examined during prenatal consultations to ensure their early detection. Such episodes should be considered a normal topic of discussion, for even if many patients do not initially admit they are suffering, these consultations may provide opportunities to encourage them to seek help.

The findings of this survey highlight the importance of examining mental health in light of stressful events experienced during pregnancy and the postpartum period. In this context, the occurrence of marital separation or divorce in the last year showed a confirmed association with PPD in the multivariate analysis. After eliminating confounding events, the possibility of PPD symptoms occurring was almost four-fold higher. These findings are corroborated by those of Ward et al. [35], who monitored 10,231 pregnant women for 8 years and predicted a significant increase in the probability of PPD in cases of marital separation or divorce [35]. Thus, a dramatic change in the family, such as divorce during pregnancy, seems to have a negative impact on women’s mental health. However, there is a paucity of literature on this subject, which underscores the need to include questions about stressors unique to divorce in research tools, thus facilitating the analysis of its potential consequences on the mother and unborn child.

Many questions about prenatal development remain unanswered, particularly about the consequences of stress and depression on the fetal environment. It is known that an increase in paternal PPD [36] may indicate that the hormonal feedback of the hypothalamic-pituitary-gonadal (HPG) axis and hypothalamic-pituitary-adrenal (HPA) axis are not the only components of these complex mechanisms [37], in which psychosocial [38] and genetic factors, among others, are interlinked [39]. It should be pointed out that some women may be more vulnerable to psychological postpartum events because of their atypical response to the hormonal changes that occur during pregnancy and birth [40]. Moreover, the reproductive hormones can alter the neurobiological patterns that significantly affect the mother-child relationship [41]. In fact, the HPA axis comprises cortisol, adrenocorticotropic hormone and corticotropin-releasing hormone, whose levels are altered in response to both pregnancy and stress. It is suspected that high levels of cortisol resulting from stress or pregnancy affect the mood of pregnant women [42]. This information is consistent with the findings of this study, in which the variables of “separation or divorce” and “clinical diagnosis of ZIKV infection during pregnancy,” which are considered stressors, were positively correlated with severe symptoms of PPD (Table 5). In this context, other investigations have suggested that stressful events [43–47] have serious long-term effects on mother and baby [48], and that, when experienced in the perinatal period, all such events may increase the possibility of triggering PPD symptoms [49]. It is worth mentioning that the above findings were confirmed in a cohort study, which analyzed the impact of disasters on the mental health of exposed individuals and found that the number of stressful events in life are predictors of this disorder [45].

On the other hand, it is still difficult for mothers to separate the effect of a perceived threat from the direct action of the virus in triggering PPD [50–52]. In this context, ZIKV infection emerges as a new factor, which, despite its low mortality rate among mothers, has led to severe clinical conditions that can impair reproductive organs and the central nervous system (CNS) of the fetus [53]. The first reports of newborns with ZIK virus-related malformations occurred in October 2015, after several reports of increased microcephaly in northeastern Brazil, which had spread to 27 states in the country by May 2016 [54]. In view of these events, a “Public Health Emergency” [55] and a “Public Health Emergency of International Concern” [56] were declared.

In this context, this study found that a clinical diagnosis of ZIKV infection during pregnancy was one of the factors responsible for the emergence of severe symptoms of IPPD detected in puerperal women (OR = 19.82; CI = 5.35–73.39). This finding may have been due to the confirmation of the vertical transmission of ZIKV to the fetus and its association with microcephaly [53]. Thus, it is a well known fact that, during pregnancy, the participants of this survey received alarming information about the potential problems their babies might face due to their exposure to Zika virus.

However, the greatest aggravating factor in this situation was that the interviewees clinically diagnosed in the early months of pregnancy received serological confirmation of the infection only during puerperium; in other words, they spent a large part of their pregnancy fearing that their baby might have microcephaly. In fact, the strong possibility that these pregnant women might fall ill should be seen as a public health problem, given the correlation between depression and suicide, maternal infanticide [57], early weaning [58], severe anxiety [59], late access to health services, a low number of prenatal consultations, and the fact that recommended physical examinations are often neglected [60]. Moreover, it weakens the mother-baby bond significantly [61]. Lastly, in newborns, ZIKV infection is associated with death, reduced head circumference (microcephaly) and low birth weight, as well as low Apgar scores, premature birth and congenital anomalies [62]. A possible explanation for severe fetal impairments is that pregnant women suffering from anxiety, depression and stress have high levels of cortisol that can pass through the placental barrier and worsen the fetal damage caused by ZIKV [63].

In addition to these factors, it should be pointed out that the use of ELISA for the laboratory diagnosis of Zika virus infection is an important diagnostic tool, although it is not widely available in Brazil. In addition, ELISA can generate false-positive results by cross reactions with other flaviviruses such as Dengue, which is widely distributed in tropical regions [64]. Moreover, the classic clinical symptoms of this infection are similar to those of Dengue fever and Chikungunya fever, making it difficult to diagnose clinically. In addition, the social commotion resulting from the fear of an outbreak may lead to the assumption, albeit inconsistent, that numerous diseases are caused by Zika virus [65]. Advances have been made in diagnosis, treatment and prevention strategies. Nevertheless, a clear understanding of the pathophysiology of this infection has yet to be achieved [66].

The findings of this study therefore indicated that exposure to the diagnosis of ZIKV infection, even if only clinical, had profound and possibly lasting effects on the mental health of these puerperal women, confirming its association with symptoms of PPD and exacerbating their feelings of anxiety and stress.

In view of this situation of total vulnerability, the public health authorities could implement a few interventions, such as the implementation of psychoeducational support groups aimed at coping with prenatal stress [67], including delivery [68]. Other options would be the implementation of social support programs based on information technology, on the web [69], and also on smartphone users [70]. Another suggestion is the practice of routine home visits before and after childbirth [49]. Lastly, the use of tools to screen depression during the pregnancy-puerperal cycle is recommended, as well as referral of the most critical patients to psychologists and psychiatrists.

## Limitations

Some limitations of this study are the adoption of a high cut-off point (EPDS ≥13) to avoid conflicting diagnosis, which reduced the size of the group of exposed women (*n* = 36), and the impossibility of generalizing the results to the private health sector. Although these methodological limitations did not jeopardize the relevance of this survey, additional studies are suggested.

This study addressed a unique theme and presented multiple strengths, such as the adoption of an analytical epidemiological study design, the inclusion of a large number of independent variables to evaluate possible associations, the use of an internationally recognized instrument validated in Brazil (EPDS), and a pilot study. Moreover, since this survey was conducted at the height of a pandemic, with strong emotional implications for the mother, the group selection bias and recall bias foreseen in case-control studies were minimized. Lastly, it should be noted that the sample size was calculated and was found to be representative. Although the findings of this study cannot be generalized to the private health sector, its methodological limitations do not diminish its relevance.

## Conclusions

This study is contextually distant from most others in that it provides preliminary information that expands the understanding of the complex interaction between diagnosed ZIKV infection and severe symptoms of IPPD. These findings point to the need for routine screening of these symptoms, particularly in individuals with a history of marital separation in the preceding year and a diagnosis of ZIKV infection during pregnancy. In addition, it is important to plan and implement integrated preventive measures aimed at pre- and postpartum mental health.

## Clinical relevance of this study

This article identifies several needs of the victims of postpartum depression. In particular, it offers groundbreaking information about the serious association between clinically diagnosed ZIKV infection during pregnancy and the onset of symptoms of IPPD. In this context, it makes an important contribution to the diagnosis, because it broadens the perception of multidisciplinary teams to maternal suffering, thereby facilitating treatment strategies in a new area that is still poorly understood.

## Data Availability

The datasets used and/or analyzed in this study are available from the corresponding author upon reasonable request.

## Abbreviations

AAS: Abuse Assessment Screen
ABEP: Brazilian Market Research Association
CDC: Centers for Disease Control and Prevention
CEP: Research Ethics Committee
CI: Confidence interval
CNS: Central nervous system
ELISA: Enzyme-Linked Immunosorbent Assay
EPDS: Edinburgh Postnatal Depression Scale
HPA: Hypothalamic Pituitary Adrenal axis
HPG: Hypothalamic Pituitary Gonadal axis
IPPD: Immediate Postpartum Period depression
MS: Brazilian Ministry of Health
MT: (state of) Mato Grosso
OR: Odds ratio
PPD: Postpartum depression
SD: Standard deviation
SPSS: Statistical Package for Social Sciences
UFG: Federal University of Goiás
UFMT: Federal University of Mato Grosso
USA: United States of America
VIF: Variance Inflation Factor
WHO: World Health Organization
ZIKV: Zika virus
